# The COVID-19 pandemic and wellbeing in Switzerland-worse for young people?

**DOI:** 10.1186/s13034-024-00760-w

**Published:** 2024-06-06

**Authors:** D. Gondek, L. Vandecasteele, N. Sánchez-Mira, S. Steinmetz, T. Mehmeti, M. Voorpostel

**Affiliations:** 1 FORS Swiss Centre of Expertise in the Social Sciences, c/o Université de Lausanne, room 5893, Géopolis, 1015 Lausanne, Switzerland; 2 Institute of Sociology, University of Neuchâtel, Neuchâtel, Switzerland; 3 Institute of Social Sciences (ISS), University of Lausanne, Lausanne, Switzerland

**Keywords:** Wellbeing, Life satisfaction, Positive affect, Negative affect, Young people, Trajectories, Covid-19 pandemic

## Abstract

**Background:**

The key objective of our study was to describe the population-average trajectories of wellbeing, spanning the period of 2017–2022, comparing young people with other age groups. Moreover, we aimed to identify subgroups of young people who experienced disproportionate changes in wellbeing.

**Methods:**

We used longitudinal data from six waves (2017–2022) of the Swiss Household Panel. Participants were at least 14 years old in 2017 and had at least one valid composite measure of wellbeing between 2017 and 2022 (n individuals = 11,224; n observations = 49,032). The data were typically collected with telephone or web interviewing. The age of participants ranged from 14 to 102, with a roughly equal distribution of men (51.1%) and women (48.9%). We conceptualized wellbeing as positive affect and life satisfaction, negative affect, stress and psychosomatic symptoms. We described the trajectories of wellbeing using piecewise growth curve analysis. We included sociodemographic characteristics to further describe wellbeing trajectories across subgroups of young people. These comprised (1) gender, (2) migration status, (3) partnership status, (4) living with parents, (5) education/employment status, (6) household income.

**Results:**

Young people (age 14–25) experienced a steady decline in positive affect and life satisfaction throughout the entire period, with the greatest change occurring before the pandemic (2017–2019). The trajectories in this outcome were largely stable in other age groups. Moreover, young individuals showed a more pronounced increase in negative affect, particularly in the pre-pandemic years, compared to older groups. Negative affect increased during the pandemic, followed by a subsequent decline post-pandemic, observed similarly across all age groups. Among young people specifically, the trajectory of stress was similar to the one of negative affect. However, issues such as sleep problems, weakness, weariness, and headaches continued to increase in this population from 2017 to 2022. We also found evidence for a greater increase in negative affect during the pandemic in young women and those not in employment or education.

**Conclusions:**

Given the fact that the decline in young people’s wellbeing in Switzerland started two years before the pandemic, our study emphasises the importance of consideing their wellbeing within a broader systemic context beyond pandemic-related changes.

**Supplementary Information:**

The online version contains supplementary material available at 10.1186/s13034-024-00760-w.

## Introduction

### Mental health and wellbeing before and during the Covid-19 pandemic–young people

There has been an enormous amount of evidence on the effects of the Covid-19 pandemic on wellbeing and mental health. Overall, the evidence suggests that mental health and wellbeing declined to some extent during the pandemic in Western countries [1–4]. However, these changes are typically described as “small” in magnitude, with most of the population showing resiliency [1–4]. For instance, a systematic review of 137 distinct studies involving 134 cohorts revealed no changes in general mental health or anxiety symptoms, but depression symptoms worsened minimally during the pandemic compared with pre-pandemic (2018–2019) [3]. However, large inconsistencies persist in the evidence, with studies showing negative, null, or even positive effects of the pandemic [1–4]. This may be due to differential timing of measurement, sociodemographic characteristics of the population, definition and measurement of mental health problems [5].

There is some evidence that that mental health and wellbeing among young people have been declining already before the pandemic [6]. However, studies of longer trends often rely on repeated cross-sectional analysis, which makes it challenging to disentangle between and within person effects [6]. For instance, a large yearly cross-sectional survey of US teenagers (*N* = 1,260,159) showed a gradual increase in depressive symptoms between 2012 and 2018, particularly among girls [7]. These changes seemed universal across different ethnic and socioeconomic groups [7]. There are few studies that include longer pre-pandemic trends as well as changes during the pandemic. For instance, one analysis of the UK Household Longitudinal Study found that psychological distress has been already increasing since 2014 [8].

The most consistent finding emerging from reviews is that mental health and wellbeing of young people were affected to a greater extent than that of the general population [1–4]. In Switzerland, young people (under 25-years-old) experienced lower life satisfaction and higher negative affect compared to pre-pandemic levels, despite no pre- vs. during pandemic differences across all ages combined [9, 10]. A longitudinal survey of over 1000 Swiss young adults (with average age around 20 years) also indicated greater levels of depression symptoms and anxiety in 2021 compared with 2018 [11]. Another prospective-longitudinal study, including 22 years-olds at the time of the pandemic from the area of Zurich, found increased average levels of perceived stress and anger, but not internalizing symptoms during the pandemic compared to before [12]. Young people may not be equipped with skills to deal with stressors of the pandemic, such as severe disease or death, worry about health [13, 14]. Adolescence and young adulthood are critical developmental stages, characterized by transitions across multiple life domains [15]. These transitions could be especially difficult during the pandemic, translating into a greater decline in wellbeing in this age group. Therefore, mental health of young people has been high on the political agenda both internationally and in Switzerland [16, 17].

Hence, the key objective of our study was to describe population-average trajectories of wellbeing among young people in 2017–2022, comparing them to older adults, in Switzerland. We aimed to produce more representative values on wellbeing in the Swiss context, than previous studies, and on a longer time trend. In our study, we broadly conceptualized wellbeing as how people feel, how they function both on a personal and social level, and how they evaluate their lives [18]. In line with this definition, we included indicators of subjective wellbeing, following the framework proposed by Diener [19], capturing positive affect, life satisfaction and negative affect. In addition, we examined trajectories in less frequently studied outcomes - stress and psychosomatic symptoms. For brevity, we limited reporting about the trajectories of stress and psychosomatic symptoms to the younger age group (14–25 years old). This was due to this population being the key interest of our project, the Covid Generation [20], and the possibility that these indicators are likely to be non-invariant across age, particularly psychosomatic symptoms. This is due to evidence that older people tend to value psychosocial resources more highly than physical functioning, which is expected to decline with age. Hence, older individuals with levels of wellbeing comparable to their younger counterparts may report worse psychosomatic symptoms, such as headaches or weakness [21]. This may make age comparisons problematic. Our secondary objective was to identify groups of young individuals particularly vulnerable during the observation period, with the key focus on the pandemic, using typically available sociodemographic indicators. This was based on the previous research indicating large heterogeneity in the extent to which various population groups were affected by the pandemic [1–4, 22]. For instance, women, socioeconomically disadvantaged individuals, or migrants have been found to have disproportionately higher psychological distress during the pandemic, however the findings were not always consistent [1–4].

### Covid-19 pandemic in Switzerland–context

The first case of infection in Switzerland was confirmed on 25 February 2020. Most educational institutions and shops were closed in Switzerland on 16 March 2020, with public gatherings including more than five people being banned on 20 March [23]. Individuals were recommended to stay at home, while outdoor activities were allowed in groups up to five with adequate physical distance. Despite efforts to reduce the spread of the virus, intensive care units were close to full occupancy by the end of March. A gradual easing of preventive measures began at the end of April, with a complete opening on 8 June 2020, but new measures were imposed in October as cases surged again. The vaccination campaign started in Switzerland on 23 December 2020, reaching a vaccination rate of 69% by February 2022. From 13 September 2021 until 17 February 2022 access to indoor public spaces was only permitted with a valid Covid certificate. The pandemic had a significant impact on the economy, with a record high decline of the gross domestic product by 10.5% in the first six months of 2020, while roughly 30,000 individuals lost their jobs in March and April, resulting in the rise of unemployment nearly as high as in all of 2010 after the financial crisis [24]. Nonetheless, Switzerland suffered the consequences of the Covid-19 pandemic to a lesser degree than most other European countries. For instance, the gross domestic product declined by 6% more on average in the European Union than in Switzerland [25]. In addition, as opposed to some European countries, such as Italy or Spain, no strict confinements were introduced in Switzerland [26]. Hence, the “milder” lockdown may have had less of an impact on mental health than in other countries.

## Materials and methods

### Data

This paper draws on longitudinal data from six waves (2017–2022) of the Swiss Household Panel [27]. Despite the SHP starting already in 1999, we included only waves between 2017 and 2022. The reason for this was that the key interest of the study was the within-person change in wellbeing around the time of the Covid-19 pandemic. Hence, we aimed at selecting a well-defined sample of participants who have gone through the pandemic and were eligible to participate during the entire observation period. Examining the trend spanning all available years of 1999–2022 could lead to conflating between and within person effects and producing distorted estimates due to inclusion of refreshment samples. We included six waves of data, as panel members who stopped participating because of health- or age-related problems contribute on average 5.4 ± 3.5 years [28].

The SHP is a nationally representative household-based panel study that collects information yearly on different aspects of life from each household member at the time of the interview [27]. After the initial sample that started in 1999, refreshment samples were added in 2004, 2013 and 2020. Every household member aged at least 14 is eligible to answer to the individual questionnaire.

On average, participants contributed 4.4 responses, ranging from one to six and with 29.0% having no missing values of wellbeing. Over half of the participants had two or fewer missing well being values. The characteristics associated with having missing information in our study were similar to those documented for overall attrition in the Swiss Household Panel [28]. That is, men, younger individuals, migrants, those with lower wellbeing and lower household income were more likely to have missing data. Hence, the sample included in our study may be under-representative for individuals with these demographic characteristics. 

The data collection period for each study wave was relatively spread out, typically ranging from September to March, with around 90% of the interviews conducted by November. We define our sample as those who were at least 14 years old in 2017, were eligible to participate in all waves between 2017 and 2022 (hence not including refreshment sample from 2020) and had at least one valid composite measure of wellbeing between 2017 and 2022 (n individuals = 11,224; n observations = 49,032; n household = 11,853, where each household has a unique id at each wave).

The individual questionnaire was administered mainly by Computer Assisted Telephone Interviewing (CATI) (> 95% for waves 2017–2019, and about 75% for 2020–2022), with Computer Assisted Web Interviewing (CAWI) used for less than 5% prior to 2020 and about 25% after. The difference in modes over time is due to the start of a mixed-mode refreshment sample in 2020 with a 50/50 division between CATI and CAWI. Only few participants changed survey mode over time. Very few participants were interviewed through Computer Assisted Personal Interviewing (CAPI) (< 0.4%).

### Measures

#### Subjective wellbeing

As the SHP does not include a validated battery measuring subjective wellbeing, we selected items that measured various aspects of life satisfaction, positive and negative affect, which were consistently present across all survey waves from 2017 to 2022 (described in more detail in Table 1). The response scale of each item ranged from 0 to 10. A higher score indicated greater satisfaction in each life domain and more frequent positive and negative affect. As we found evidence for the items to fall on separate factors, we derived two components of wellbeing–positive affect and life satisfaction (PALS) and negative affect (NA)–by summing up the relevant items (as indicated in Table 1) [29]. These two subscales were found to have high internal consistency (Cronbach’s alpha ranging 0.75–0.78) and be scalarly invariant across gender and time, meaning that the questions are likely to be equivalently interpreted across those groups [29]. PALS and negative affect were moderately negatively correlated (Pearson’s *r* = − 0.39).

#### Stress and psychosomatic symptoms

Stress was measured as a subjective evaluation of whether participants suffered from stress in the last month (0– “never” to 5– “very often”). It was treated as a continuous variable. Stress correlated weakly negatively with PALS (*r* = − 0.24) and moderately positively with negative affect (*r* = 0.42).

Respondents were asked to indicate whether they suffered from psychosomatic symptoms– including sleeping problems, headaches, and weakness or weariness– “not at all”, “somewhat” or “very much” in the last four weeks. These indicators were recoded into binary variables (0 = “not at all” vs 1=”somewhat/very much”). Tetrachoric correlation between individual symptoms was weak ranging from 0.08 to 0.26.

**Table 1:** Details about the measure of wellbeing

Wellbeing domain	Questions	Response options	Additional details
Positive affect and life satisfaction (PALS)	**Life satisfaction**: In general, how satisfied are you with your life?	0 (not at all satisfied)–10 (completely satisfied)	Positive affect and life satisfaction was derived by summing up the items, hence ranging from 0 to 60, with a higher score showing greater frequency of negative affect Cronbach’s Alpha (α) = 0.75–0.76 across years
	**Health satisfaction**: How satisfied are you with your state of health?	0 (not at all satisfied)–10 (completely satisfied)	
	**Relationships satisfaction**: How satisfied are you with your personal, social and family relationships?	0 (not at all satisfied)–10 (completely satisfied)	
	**Leisure time satisfaction**: How satisfied are you with your leisure time activities?		
	**Energy and optimism**: Are you often plenty of strength, energy and optimism?	0 (never)–10 (always)	
	**Joy**: How frequently do you generally experience the following emotions?	0 (never)–10 (always)	
Negative affect (NA)	**Anger**: How frequently do you generally experience the following emotions?	0 (never)–10 (always)	Negative affect was derived by summing up the items, hence ranging from 0 to 40, with a higher score showing greater frequency of negative affect Cronbach’s Alpha (α) = 0.76–0.78 across years
	**Sadness**: How frequently do you generally experience the following emotions?		
	**Worry**: How frequently do you generally experience the following emotions?		
	**Anxiety and depression**: Do you often have negative feelings such as having the blues, being desperate, suffering from anxiety or depression?		
Stress	How often have you felt stressed during the last month?	1 (never)/2 (almost never)/3 (sometimes)/4 (fairly often)/5 (very often)	The variable was normally distributed, hence we treated it as continuous
Psychosomatic symptoms	**Sleeping problems**: During the last 4 weeks, have you suffered from any of the following disorders or health problems?	0 (not at all)/1 (somewhat)/2 (very much)	The proportion of „very much” option was relatively small compared to two other options (on average around 10%). Hence, we binarized the variable (0– “not at all” vs. 1– “somewhat/very much”) allowing to plot a single trajectory per variable, which made the interpretation easier
	**Headaches**: During the last 4 weeks, have you suffered from any of the following disorders or health problems?		
	**Weakness, weariness**: During the last 4 weeks, have you suffered from any of the following disorders or health problems?		

#### Sociodemographic characteristics

We included sociodemographic characteristics to further describe wellbeing trajectories across subgroups of young people. These were collected in 2019 and comprised: (1) gender (man/woman), (2) being a migrant based on the first nationality (Swiss/non-Swiss), (3) partnership status (married or living with a partner/single, not living with a partner/widowed/divorced/separated), (4) living with parents (vs. not), (5) being in education or training/employment/both/neither, (6) equivalized household net income, categorized into quartiles [30]. The age variable represents the age of participants during the pandemic in 2020.

### Analyses

All the analyses were conducted in Stata v.16 [31], code is available online at https://osf.io/vnzcw/?view_only=ffad7d69ae24416692bbdb413363f185.

#### Population-average trajectories of wellbeing–comparing 14–25-year-old to older adults

The first study objective–to describe population-average trajectories of wellbeing across different age groups in 2017–2022, with a key focus on young people– was addressed using piecewise growth curve analysis. We conducted this analysis within a multilevel modelling framework, whereby PALS, negative affect and stress were treated as continuous outcomes. We reported regression coefficients when describing the trajectories and differences in these coefficients when making age comparisons. Psychosomatic symptoms were also modelled with piecewise growth curve analysis, but using generalized linear model (GLM) due to being binary indicators, with a log link function and robust standard errors. The changes in intensity of symptoms were expressed as predicted probabilities of “somewhat” or “very much” suffering from a given symptom.

This approach accounts for the multilevel structure of longitudinal data, where occasion specific measurements are nested within individuals [32]. Hence, it recognises that responses given by the same individual over time are correlated with each other and provides more conservative standard errors [32]. Our model included both fixed effects and random effects. Fixed effects, similar to regression coefficients, represent population average effects of time. Random effects include information about variance around the starting point of the trend (an intercept) and the trend itself (a slope). As a sensitivity check, we tested whether accounting for clustering of individuals within households made any difference to our estimates. This was done by including household as third level clustering variable in the random part of the growth curve analyses. However, as we found no differences in estimates, the results are not reported.

In our study, we conceptualized time as four separate periods (slopes), representing an overall trend between 2017 and 2022—for this reason we refer to our analysis as “piecewise” growth curve modelling. The four slopes were (1) pre-pandemic—2017–2019, (2) into-pandemic—2019–2020, (3) during pandemic—2020–2021, (4) out-of-pandemic—2021–2022. Conceptualising time in this manner allows us to directly compare change in two different periods. For instance, whether *a potential* decline in wellbeing during pre-pandemic equals *a potential* improvement in wellbeing in the post-pandemic period. This can be tested using the Wald test, which formulates the null hypothesis that these two slopes equal zero (i.e., slope_2017 2019_–slope_2021−2022_ = 0).

We allowed the trajectories (or slopes) to vary by including interaction terms between the age groups and slopes (i.e., age*slope_2017−2019_, age*slope_2019−2020,_ age*slope_2020−2021_, age*slope_2021−2022_). We derived a categorical variable representing four age groups: (1) age 14–25, (2) age 26–45, (3) age 46–65, (4) age > 65.

To explicitly test whether there were any differences between the youngest group (14–25) and older groups, we ran a series of Wald tests. First, we conducted an omnibus Wald test to examine whether there were any differences across the entire study period between the age groups (i.e. a test for all interactions simultaneously: age*slope_2017−2019_, age*slope_2019−2020,_ age*slope_2020−2021_, age*slope_2021−2022_ = 0, at p value < 0.05). We also used Wald tests for the interaction at specific time periods (e.g., age*slope_2017−2019_ = 0), as there may be differential time-specific slopes across age groups not detected by the omnibus test. If evidence for an interaction was detected (at *p* < 0.05), we further investigated where the differences occurred (i.e., between which age groups and during which period) by running pairwise comparisons of estimated marginal means. All analyses controlled for survey mode. As a supplementary analyses, we conducted an analysis of all individual items, which allowed us to examine whether the average trajectories tended to be driven by single items.

#### Identifying subgroups of vulnerable young people (14–25-year-old)

Our secondary objective was to identify subgroups of young people (age 14–25) who experienced disproportionate changes in wellbeing. Using a similar approach as described in “Population-average trajectories of wellbeing–comparing 14–25-year old to older adults”, we allowed the slopes to vary across different sociodemographic groups among young people, by including interaction terms (e.g., gender*slope_2017−2019_, gender*slope_2019−2020,_ gender*slope_2020−2021_, gender*slope_2021−2022_). More details on the used covariates can be found in “Sociodemographic characteristics”. Potential differences were further explored using the Wald test and marginal effects.

#### Dealing with missing information

The detailed description of our missing data strategy is provided in the Supplementary Text 1 (including Supplementary Table 1). We used two approaches to account for missing information and to reduce a potential bias in results–multiple imputation and Maximum Likelihood (ML).

ML estimation was used for the analysis of population-average trajectories of wellbeing, psychosomatic symptoms and stress (see “Population-average trajectories of wellbeing–comparing 14–25-year old to older adults” and “Identifying subgroups of vulnerable young people (14–25-year-old)”). ML allows for fitting the trajectories including individuals with at least one measure of wellbeing and no missing covariates. Using ML in subgroups analysis with sociodemographic covariates (see “Dealing with missing information”.) would result in losing additional cases, as there was a greater amount of missing information in these variables than in wellbeing. Hence, we used multiple imputation, generating 50 samples, to replace the missing values. Both approaches provide unbiased results under the Missing at Random (MAR) assumption. As a robustness check, we also described the population-average trajectories of wellbeing using the multiply imputed sample. This was done as the MAR assumption may be likely to be met in multiple imputation due to inclusion several variables that predict wellbeing and missingness in the model.

## Results

### Descriptive information about the population

Descriptive information about the population can be found in Table 2.

**Table 2:** Demographic characteristics of the participants

	Missing *n* (%)/ non-missing *n*	*n* (% of participants in each category)
*Age*	0 (0.0)/ 11,241^1^	
14–25		1394 (12.4)
26–45		2792 (24.8)
46–65		3969 (35.3)
>65		3086 (27.5)
Young people only (14-25-year-old, mean age = 21.15, standard deviation = 2.55; total *n* = 1,394)^2^		
*Gender*	0 (0.0)/ 1394	
Men		712 (51.1)
Women		682 (48.9)
*First nationality*	0 (0.0)/ 1394	
Swiss		1272 (91.3)
Non-Swiss		122 (8.8)
*Partnership status*	234 (16.8)/ 1160	
Married or living with a partner		65 (5.6)
Single, not living with a partner		1095 (94.4)
Widowed/divorced/separated		0 (0.0)
*Living with parents*	63 (4.5)/ 1331	
No		153 (11.5)
Yes		1178 (88.5)
*In education (including training)/employment*		235 (16.9)/ 1159
Education/training		435 (37.5)
Employment		296 (25.5)
Both		384 (33.1)
Neither		44 (3.8)
*Income (quartile)*	355 (25.5)/ 1039	
1st		243 (23.4)
2nd		328 (31.6)
3rd		275 (26.5)
4th		193 (18.6)

^1^This represents the total number of participants across all ages

^2^This represents the total number of participants in the youngest age group (14–25)

### Population-average trajectories of wellbeing–comparing 14–25-year-old to older adults

**Fig. 1:**
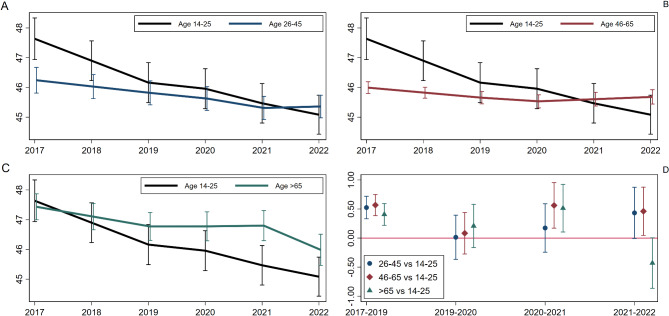
Age-specific average trajectories of positive affect and life satisfaction (panels **A**–**C**) and comparison of period-specific difference in change across age groups, with young people as a reference group (panel **D**)

As can be seen in Fig. 1 (panels A-C) young people (age 14–25) experienced a steady decline in wellbeing over the entire period, with the greatest change before the pandemic (2017–2019). Their PALS score dropped by between 0.41 (0.22 to 0.59) and 0.52 (0.33 to 0.72) compared to older groups (see Fig. 1–panel D). The youngest group also experienced the greatest decline in PALS during the pandemic (2020–2021) (by 0.56, 0.17 to 0.96 more than age 45–65 and 0.51, 0.10 to 0.92 than age > 65). However, the decline among the youngest was not greater during the pandemic than before, so it appears to be a continuation of a longer trend. Finally, during the out-of-pandemic (2021–2022) period the decline of wellbeing among young people continued. The trajectories were stable among 26–45 and 46–65-year-olds but declined to a greater extent among the oldest group (by − 0.43, − 0.86 to 0.00), albeit starting from a higher level.

**Fig. 2:**
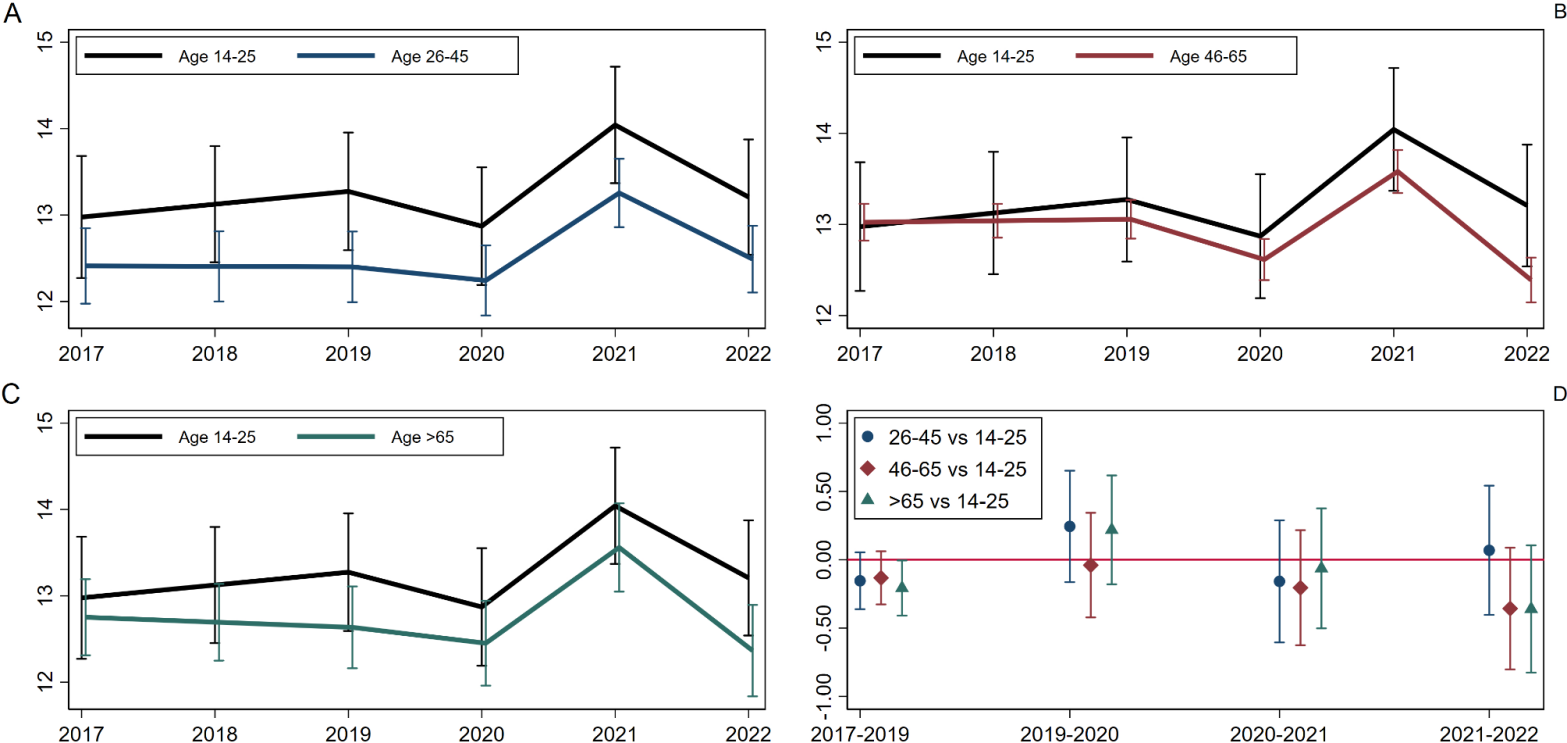
Age-specific average trajectories of negative affect (panels **A**–**C**) and comparison of period-specific difference in change across age groups, with young people as a reference group (panel **D**)

With respect to negative affect, young people experienced an increase during the pre-pandemic period (2017–2019). However, there was a considerable uncertainty around the estimates as reflected by wide confidence intervals (0.13, − 0.04 to 0.29 a year) (see Fig. 2–panels A-C). This increase was greater than among older people, by 0.13 (− 0.06 to 0.33) compared with age 45–65 and 0.21 (0.01 to 0.41) compared with age 65 or older.

We did not detect any age differences in the trajectories of negative affect for the into-pandemic period (see Fig. 2–panel D). During this time (2019–2020) the levels of negative affect declined among the youngest group (age 14–25) (− 0.45, − 0.78 to − 0.12), and midlife individuals (age 46–65) (− 0.44, − 0.61 to − 0.26), whereas for two other age groups the estimates were largely imprecise (age 26–45: − 0.16, − 0.37 to 0.04; age > 65: − 0.16, − 0.39 to 0.07). Also, the increase in negative affect during the pandemic (2020–2021) was comparable across all age groups, with the youngest group experiencing a rise of 1.14 (0.77 to 1.50) (see Fig. 2–panel D). Subsequently (2021–2022), we saw a decrease in negative affect across all age groups, with the 14–25 and 26-45-year-olds having somewhat smaller declines (age 14–25: − 0.86, − 1.25 to − 0.47, age 26–45: −0.77, − 0.99 to − 0.54 vs. age 46–65: − 1.18, − 1.38 to − 0.99, age > 65: − 1.19, − 1.46 to − 0.92). The out-of-pandemic decline largely but not completely compensated for the increase during the pandemic among young people. The out-of-pandemic decline was statistically equivalent for 45–65 and > 65-year-olds.

The age-specific trajectories of individual items can be found in the Supplementary Text 2 and Supplementary Fig. 1). The key findings were that young people experienced the greatest drop in life satisfaction during the entire study period. The decline in satisfaction with leisure activities was most pronounced among young individuals during the pre-pandemic (2017–2019) and the into-pandemic period (2019–2020). Moreover, the youngest cohort also reported a more significant rise in feelings of depression and anxiety both during the pre-pandemic (2017–2019) and pandemic period (2020–2021), along with increased levels of worry before the pandemic.

### Population-average trajectories of psychosomatic symptoms and stress among young people (14–25-year-old)

**Fig. 3:**
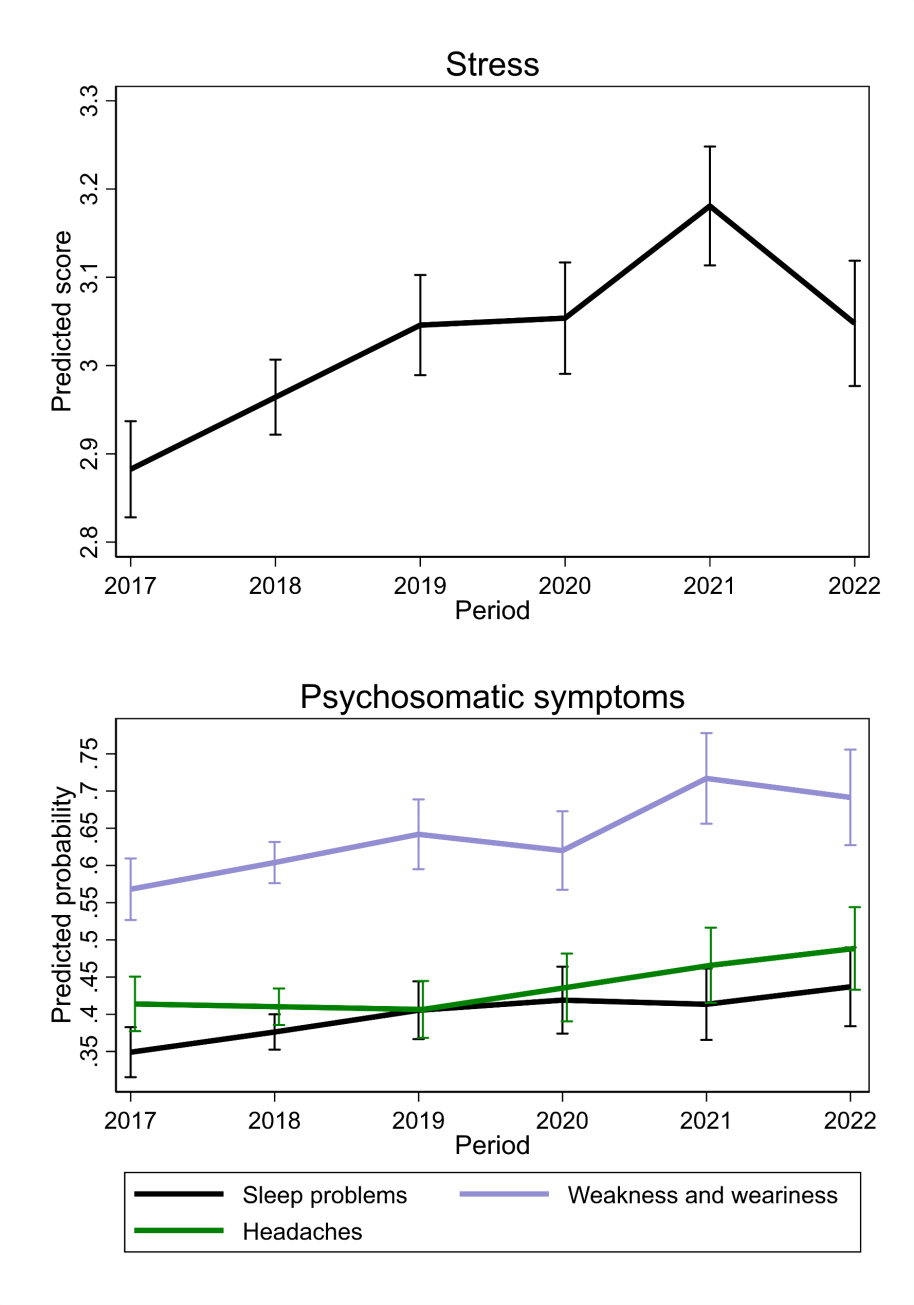
Average trajectories of stress and psychosomatic symptoms among young people (age 14–25)

Figure 3 shows that the frequency of stress increased already before the pandemic (2017–2019) by 0.08 annually (0.05 to 0.12) among young individuals. It remained relatively stable during the initial phase of the pandemic (0.00, − 0.06 to 0.08), rose once again during the pandemic (2020–2021) by 0.13 (0.05 to 0.21), and subsequently decreased during the out-of-pandemic period (2021–2022) by − 0.13 (− 0.22 to − 0.05).

As for psychosomatic symptoms, the predicted probability of individuals reporting sleep problems increased throughout the entire period from 34.9% (31.6 to 38.3) in 2017 to 43.7%, (38.4 to 49.0) in 2022, with the greatest rise before the pandemic (from 34.9%, 31.6 to 38.3 in 2017 to 40.6%, 36.7 to 44.4 in 2019). Likewise, the probability of experiencing weakness and weariness increased pre-pandemic (56.8%, 52.7 to 61.0 in 2017 vs. 64.2%, 59.5 to 68.9 in 2019), and during the pandemic (62.0%, 56.7 to 67.3 in 2020 vs. 71.7%, 65.6 to 77.8 in 2021). The probability of reporting headaches remained stable pre-pandemic but increased afterwards from 40.7% (36.8 to 44.5) in 2019 to 48.9% (43.3 to 54.4) in 2022 (see Fig. 3).

### Identifying subgroups of vulnerable young people (14–25-year-old)

They key findings were for differential trajectories in negative affect across genders and being in employment or education. The levels of negative affect declined among young women into-pandemic (2019–2020), while they stayed stable among young men (0.06, − 0.52 to 0.65 vs. − 0.76, − 1.34 to − 0.18, *p* = 0.03). Subsequently, there was a greater increase among women than men during the pandemic (2020–2021) (1.79, 1.23 to 2.34 vs. 0.77, 0.19 to 1.34, *p* = 0.04). Moreover, during the pandemic (2020–2021), young people who were neither in education nor employment experienced a more substantial rise in negative affect, reporting 3.03 (0.86 to 5.19), compared to other groups such as those in education, which showed an increase of 1.09 (0.31 to 1.86). Supplementary Table 2 includes p values of Wald tests examining interactions across all outcomes and potential effect modifiers.

## Discussion

### Key findings, previous literature, implications

This is the first study, representative of households in Switzerland, that aimed to compare the population-average trajectories of wellbeing between young people and other age groups, spanning the period of 2017–2022. Positive affect and life satisfaction decreased among young people (age 14–25) over the entire study period (2017–2022), with the greatest declines before the pandemic, which were larger than in other age groups during the entire observation period. Young people experienced a steady decline in satisfaction with life in general, and with leisure activities before and going into-pandemic. Negative affect had been showing a slight increase among young individuals before the pandemic. During the pandemic, negative affect increased and subsequently declined out-of-pandemic. This decrease was more modest among young people compared with the oldest groups, not fully compensating for the prior increase. Overall, wellbeing during the pandemic decreased in all age groups, but the decline has been observed already pre-pandemic among the youngest. This is consistent with international evidence [6–8]. In Switzerland, this has also been observed in a consistent rise in admissions to mental health services among young individuals, especially women, starting as early as 2012 [33]. These findings are consistent with studies from other Western European countries. The strength of our study in this context is that, as opposed to most previous studies, we examined changes within the same individuals [8].

Based on the existing knowledge, we can only speculate on the causes behind the decline in wellbeing and mental health among young individuals in Switzerland and other Western European countries, which began several years prior to the pandemic [6–8]. It is often suggested that young people have become more open about their mental health problems, due to greater mental health awareness [34]. This might imply that wellbeing measures do not consistently capture the same concept over time. However, like others, we found statistical measurement invariance of the wellbeing measure, indicating that the interpratation of the questions remained consistent over time [35]. Another explanation frequently offered in the literature is about the harmful effect of widespread use of social media. However, the evidence that social media may contribute to poorer wellbeing among young people is merely tentative for the time being [6, 36, 37]. Morever, others speculated that the increasingly challenging economic circumstances faced by young individuals (e.g., housing expenses, inflation) might be an important determinant of declining wellbeing. Nevertheless, in Switzerland, economic indicators have remained relatively stable over the past decade (e.g., concerning youth unemployment, youth poverty, or the growth of the gross domestic product; GDP) [38]. This was not entirely the case during the pandemic, as the GDP experienced a decline of 2.4%, and young individuals (< 25 years) were more affected by unemployment compared to other age groups. However, both the GDP and youth employment swiftly rebounded to pre-pandemic levels [38, 39]. Other potential contributors to decreased wellbeing could be the uncertainty that young people face, in terms of precarious employment, climate change and military conflicts. Young individuals might not have developed adequate coping mechanisms to deal with these challenges. This situation could be intensified by constant exposure to a vast amount of information. Indeed, studies during the pandemic have revealed that the rise in time spent on social media platforms was linked to increased symptoms of anxiety and depression [40]. The fact that wellbeing has been already deteriorating for an extended period emphasises the need for primary prevention programmes that go beyond treatment and interventions when a crisis, such as the Covid-19 pandemic, occurs [6]. Such programmes would equip youth with tools to navigate the challenges of the modern world, including social-emotional skills, peer-support, sense of connection and meaning-making [6].

Also, largely in line with previous international research, wellbeing declined to a greater extent among young people during the pandemic [1–4]. As shown previously, at least in terms of negative affect, after the initial drop, wellbeing started to improve again after the pandemic at the population level [1–4]. Providing a more holistic picture of how well young people did during the pandemic, across a comprehensive set of indicators can help to speculate about determinants of wellbeing. Stress, sleep problems, weakness and weariness all increased pre-pandemic, while the probability of headaches remained stable. Stress, weakness and weariness, and headaches increased during the pandemic, but only stress declined afterwards. The increase in psychosomatic symptoms during the pandemic was documented previously in other countries [41]. Evidence on pre-pandemic trajectories in these indicators is limited. The decline in PALS, negative affect and particularly increase of psychosomatic symptoms during the pandemic may be due to the pandemic constituting a traumatic event (or stressor) [13, 14]. It has been argued that young people may be susceptible to trauma, as the pandemic might be the first exposure to severe disease, potential death and grief for many of the young people in high income countries. This is combined with anxiety and worries about infection of themselves, friends and family as well as feelings of uncertainty, and perception of the world as scary and unsafe. Moreover, young people tended to report loneliness, isolation, concerns about education, breakdown of routines as being particularly stressful [5]. Disruption of daily activities and stress related to pandemic might have led to increased family tensions, particularly affecting young people. Also, young people could not rely on their social network during the pandemic, due to limited opportunities for socialising with their friends and extended family [42]. Adolescence and young adulthood are critical developmental stages, characterized by transitions across multiple life domains [15]. These transitions could be especially difficult during the pandemic, translating into a greater decline in wellbeing in this age group. However, as shown by multiple studies of both mass trauma and post-traumatic stress disorders most people are resilient in the mid to long term (around 55–85%) [43]. This has also been seen in our study in the trajectory of negative affect, when after the initial increase it bounced back nearly to pre-pandemic levels.

As the secondary objective, we aimed to identify subgroups who experienced disproportionate changes in wellbeing, stress or psychosomatic symptoms. We did not find any differences according to pre-pandemic characteristics, such as household income, partnership status, being a migrant, or living with parents. The literature on the changes in wellbeing during the pandemic has been largely inconsistent regarding sociodemographic differences [1–4]. However, women, migrants and socioeconomically disadvantaged individuals have often been identified as particularly vulnerable [1–4]. We only found greater increases in negative affect among women and those in neither in education nor employment/training. Those in not in education, employment, or training (NEET) may have been at a greater risk of being disconnected from opportunities or social networks typically associated with education or employment. Somewhat suprisingly, we did not find any differences according to the household income. One potential explanation is that the protective social welfare policies were largely effective for wellbeing. Likewise, we did not detect any differential changes in wellbeing according to migration or partnerhsip status and whether the pariticipants lived with their parents. This is not to say that absolute differences in wellbeing do not exist between these groups, but rather, they were not exacerbated or reduced by the pandemic.

### Strengths and limitations

The key strength of our study is that it is based on a representative sample of households in Switzerland, followed by two years pre- and post-pandemic. However, as with all longitudinal studies, attrition and nonresponse may have introduced a survival bias to the findings. Those with high wellbeing could be more likely to remain in the study, leading to an underestimation of the drop in wellbeing during the pandemic. We attempted to correct for this bias by retaining those with at least one observation during the entire study period (2017–2022) and using techniques, such as ML and multiple imputation which allow for missing data. However, this still does not correct for the bias due to not contributing any observation at all (e.g., due to attrition prior to 2017). It is possible that our estimates are more favourable than trends in general population, as younger individuals, and those more vulnerable were more likely to have missing information. That is, one could expect sharper declines in wellbeing over time, particularly during the pandemic.

The second limitation of our study is that we did not have access to a standardized measure of wellbeing. Instead, we derived it using a range of individual items capturing wellbeing. Prior to this study, we found that the measure has robust psychometric properties (e.g., a clear factorial structure, measurement invariance across time and demographic groups). However, the evaluation of effect sizes or comparisons with other studies were somewhat impeded due to the absence of a widely recognized wellbeing measure.

## Conclusion

Wellbeing of young people started to decline at least two years before the pandemic in Switzerland. Negative affect, stress and some psychosomatic symptoms increased during the pandemic and then largely bounced back to pre-pandemic levels, however still maintaining an overall rising trajectory. Hence, there is a need to consider wellbeing of young people through a wider systemic context, beyond the periodic change associated with the pandemic. At the same time, the potential impact of the pandemic should not be underestimated. Even short-lasting effects can have a large social and economic impact at the population level, for instance, by increased vulnerability to future mental health problems. Finally, young women and socioeconomically disadvantaged individuals could be more vulnerable than others and may require more targeted approaches.

## Electronic supplementary material

Below is the link to the electronic supplementary material.

Supplementary Material 1

## Data Availability

SHP data are freely accessible to the scientific community here: https://www.swissubase.ch/en/catalogue/studies/6097/19347/overview.
