# Correction to: The COVID-19 pandemic and wellbeing in Switzerland-worse for young people?

**DOI:** 10.1186/s13034-024-00816-x

**Published:** 2024-10-24

**Authors:** D. Gondek, L. Vandecasteele, N. Sánchez-Mira, S. Steinmetz, T. Mehmeti, M. Voorpostel

**Affiliations:** 1 FORS Swiss Centre of Expertise in the Social Sciences, c/o Université de Lausanne, room 5893, Géopolis, 1015 Lausanne, Switzerland; 2 Institute of Sociology, University of Neuchâtel, Neuchâtel, Switzerland; 3 Institute of Social Sciences (ISS), University of Lausanne, Lausanne, Switzerland

**Correction: Child and Adolescent Psychiatry and Mental Health (2024) 18:67**

10.1186/s13034-024-00760-w

Following publication of the original article [1], the author identified the errors in the quality of figures, incorrect captions of Figs. 1 and 2 and textual errors in Results section. These corrections have been updated with this erratum.

Figures 1 and 2 were labelled incorrectly. Figure 1  presents results for positive affect and life satisfaction and Fig. 2  presents results for negative affect. Hence, the labels should be reversed.

Figure 1: “Age-specific average trajectories of positive affect and life satisfaction (panels A–C) and comparison of period-specific difference in change across age groups, with young people as a reference group (panel D)”

Figure 2: “Age-specific average trajectories of negative affect (panels A–C) and comparison of period-specific difference in change across age groups, with young people as a reference group (panel D)”

In Results section under the sub-heading “Population-average trajectories of psychosomatic symptoms and stress among young people (14–25-year-old)”, there were also minor topographical errors related to misplaced bracket opening and the word “vs.”. The paragraph originally appeared as follows:

“As for psychosomatic symptoms, the predicted probability of individuals reporting sleep problems increased throughout the entire period from (34.9%, 31.6 to 38.3) in 2017 to (43.7%, 38.4 to 49.0) in 2022, with the greatest rise before the pandemic (from 34.9%, 31.6 to 38.3 in 2017 to 40.6%, 36.7 to 44.4 in 2019). Likewise, the probability of experiencing weakness and weariness increased pre-pandemic (56.8%, 52.7 to 61.0 in 2017 to 64.2%, 59.5 vs. 68.9 in 2019), and during the pandemic 62.0%, 56.7 to 67.3 in 2020 vs. 71.7%, 65.6 to 77.8 in 2021).”

The paragraph should appear as follows:

“As for psychosomatic symptoms, the predicted probability of individuals reporting sleep problems increased throughout the entire period from 34.9% (31.6 to 38.3) in 2017 to 43.7%, (38.4 to 49.0) in 2022, with the greatest rise before the pandemic (from 34.9%, 31.6 to 38.3 in 2017 to 40.6%, 36.7 to 44.4 in 2019). Likewise, the probability of experiencing weakness and weariness increased pre-pandemic (56.8%, 52.7 to 61.0 in 2017 vs. 64.2%, 59.5 to 68.9 in 2019), and during the pandemic (62.0%, 56.7 to 67.3 in 2020 vs. 71.7%, 65.6 to 77.8 in 2021).”

 The high resolution images (Figs. 1, 2, 3) have been given below:

**Fig. 1:**
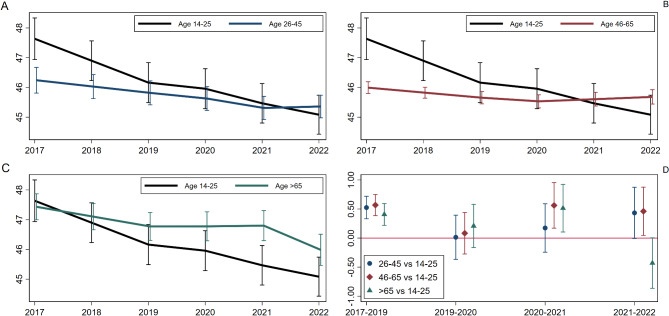
Age-specific average trajectories of positive affect and life satisfaction (panels **A**–**C**) and comparison of period-specific difference in change across age groups, with young people as a reference group (panel **D**)

**Fig. 2:**
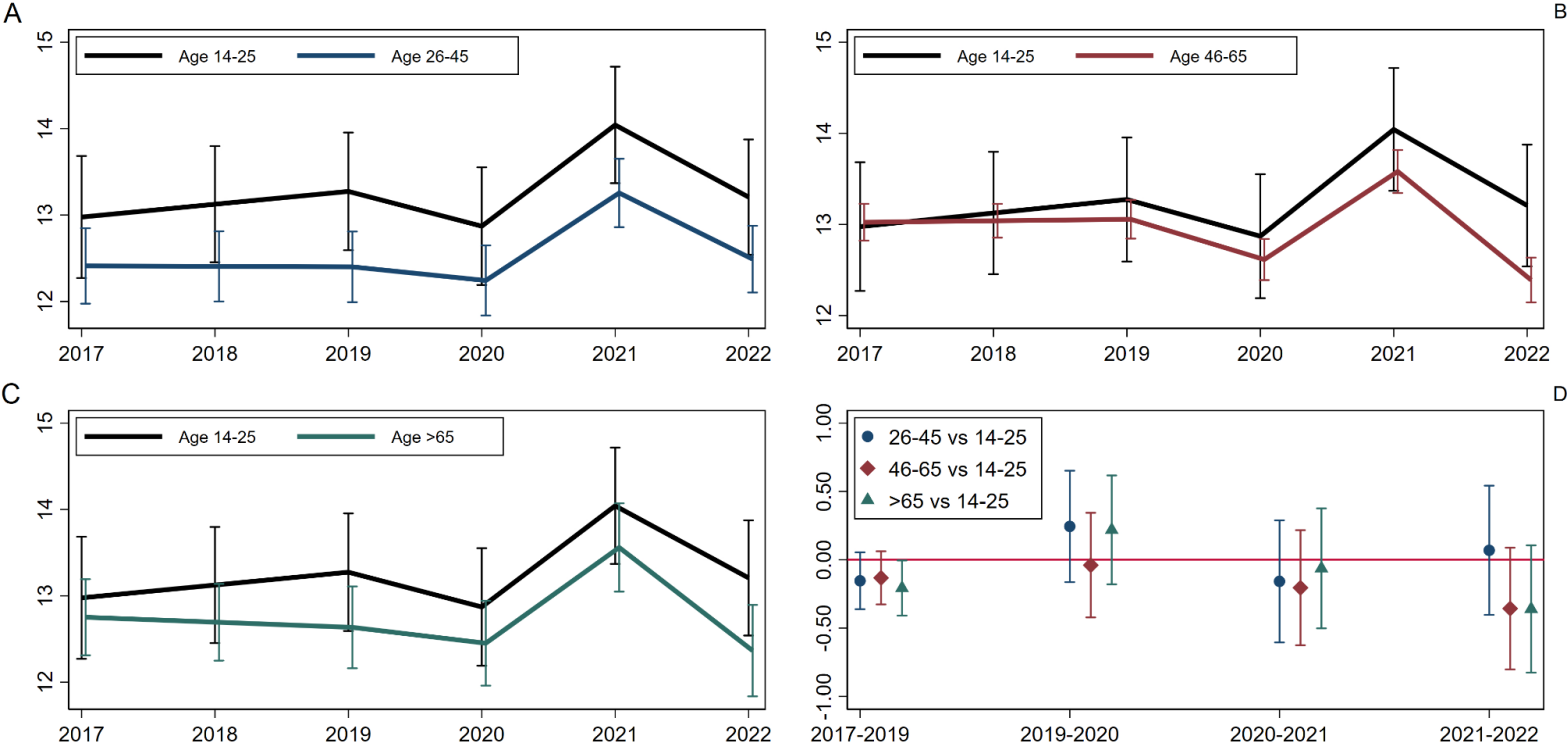
Age-specific average trajectories of negative affect (panels **A**–**C**) and comparison of period-specific difference in change across age groups, with young people as a reference group (panel **D**)

**Fig. 3:**
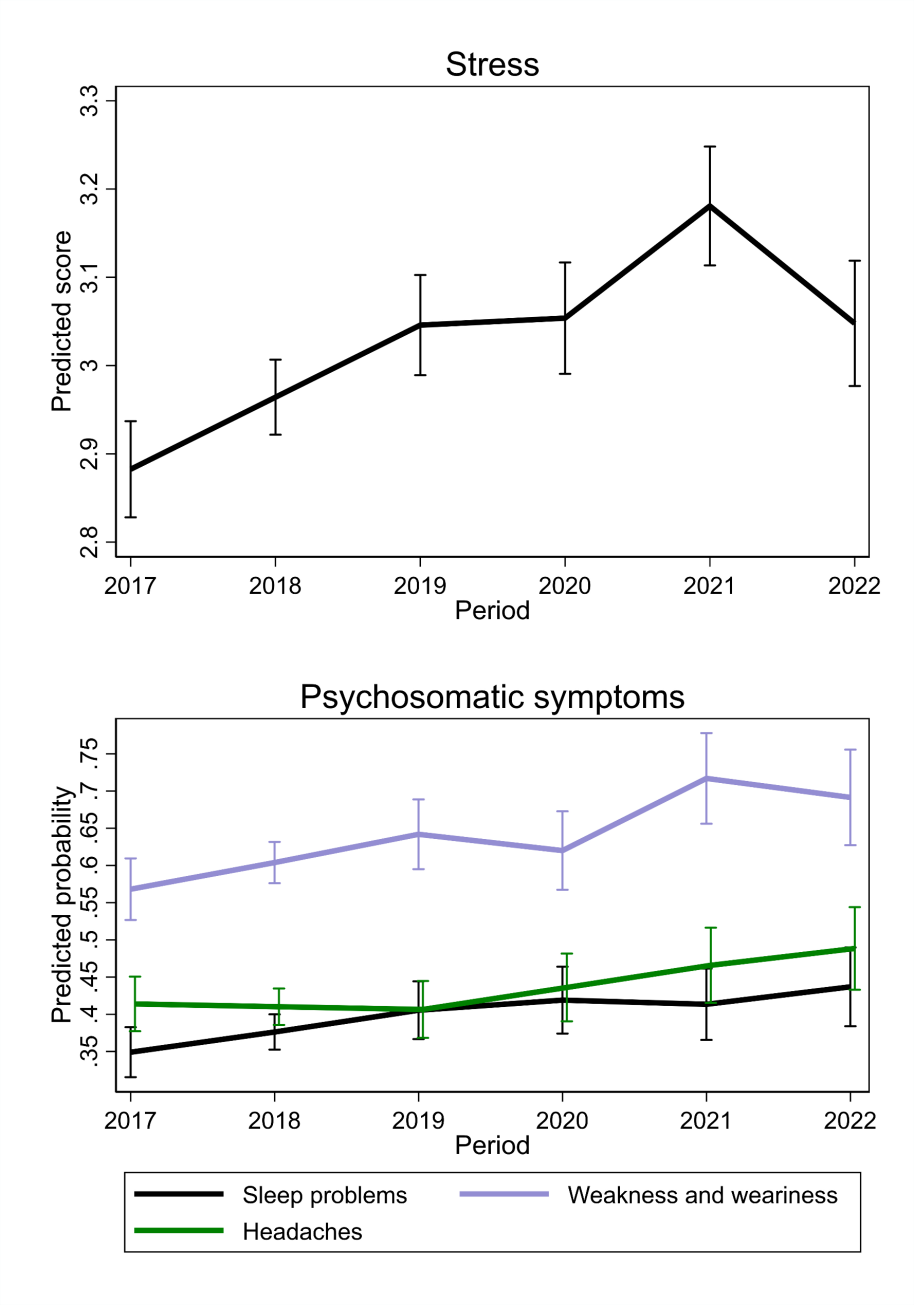
Average trajectories of stress and psychosomatic symptoms among young people (age 14–25)

The original article has been corrected.
