# Supplementary material for: The COVID-19 pandemic and wellbeing in Switzerland-worse for young people?
Source: Child Adolesc Psychiatry Ment Health. 2024 Jun 6;18:67. doi: 10.1186/s13034-024-00760-w (PMC11157761; doi:10.1186/s13034-024-00760-w)
Supplement: Supplementary file 1 — Supplementary Material 1 [file 13034_2024_760_MOESM1_ESM.docx]

# Supplementary material

Supplementary Text 1. Missing data strategy.

Supplementary Text 2: Trajectories of individual indicators of wellbeing across different age groups.

Supplementary Figure 1. Age-specific average trajectories of individual indicators of wellbeing.

Supplementary Table 1. P-values of Wald test of differential trajectories across population subgroups.

**Supplementary Text 1. Missing data strategy.**

We used two approaches to account for missing information and to reduce a potential bias in results – multiple imputation and Maximum Likelihood (ML). ML estimation was used for the analysis of population-average trajectories of wellbeing, psychosomatic symptoms and stress (see sections 2.4.1. and 2.4.2 of the manuscript). Using ML within multilevel structures allows for estimation with unbalanced data. In other words, it is not necessary to have the same number of observations (level 1) per individual participant (level 2). Hence, all available data can be incorporated into the analysis. ML works under the assumption that the data are missing at random (MAR) [2, 3]. The MAR mechanism, which is largely untestable, implies that systematic differences between the missing and the observed values can be explained by observed data [2]. This assumption may be more likely to be met when the model includes additional information (i.e., auxiliary variables) that predicts missingness and/or wellbeing. Inclusion of such variables is possible in multiple imputation; hence we reran the analysis based on the multiply imputed data, producing findings consistent to those with ML.

Multiple imputation was also used for the supplementary subgroups analysis using sociodemographic covariates (see section 2.4.3. of the manuscript). As there is missing information in these covariates that cannot be accounted for by ML, using multiple imputation allowed for including a more complete sample comprising the same participants across all analyses. The missing data were imputed in a wide format using multiple imputation by chained equations (MICE), due to the non-monotone pattern of missing values, and due to its ability to accommodate various types of variables in the imputation model, including continuous and categorical ones. This approach uses a series of univariate conditional imputation models to impute missing data [4]. The imputation models included wellbeing (positive affect and life satisfaction as well as negative affect), stress, psychosomatic symptoms and all sociodemographic covariates (gender, age, migration status, partnership status, living with parents, household income). We also included home ownership as an auxiliary variable to maximise the MAR assumption and improve the accuracy of the imputation model minimising non-random variation in the imputed values [5, 6].

**Supplementary Text 2: Trajectories of individual indicators of wellbeing across different age groups.**

In terms of indicators of PALS, we found statistical evidence for differential trajectories across age groups, as indicated by an omnibus Wald test (i.e., age*slope_2017-2019_, age*slope_2019-2020,_ age*slope_2020-2021_, age*slope_2021-2022_ = 0) in life satisfaction (p<0.0001), health satisfaction (p<0.0001), satisfaction with relationships (p<0.0001), satisfaction with leisure activities (p<0.0001), joy (p<0.0001), energy and optimism (p=0.01) (see Supplementary Figure 1).

Young people experienced the greatest drop in life satisfaction during 2017-2019 and the entire study period. Moreover, during the pandemic they became the group with the lowest satisfaction. The decline was particularly stark in comparison with the oldest group during the into-pandemic period (2019-2020) (age 14-25 vs >65: 0.11, 0.02 to 0.21).

Health satisfaction was highest among the youngest group during the entire period, however it declined to a greater extent than in other age groups, particularly during the pre- (2017-2019), and pandemic periods (2020-2021). Satisfaction with relationships declined most among the youngest group pre-pandemic (e.g., age 14-25 vs 26-45: 0.11, 0.05 to 0.16). While during pandemic (2020-2021) it was relatively stable among the youngest, it declined most in the age 26-45 and improved in age >65. A reversed effect was seen out-of-pandemic (2021-2022), when it got worse among the oldest and improved in the age 26-45, with stable values in the age 14-25.

Satisfaction with leisure activities declined the most among young people pre-pandemic (2017-2019) (e.g., age 14-25 vs >65: 0.10, 0.03 to 0.17) and into-pandemic (2019-2020) (e.g., age 14-25 vs >65: 0.14, -0.002 to 0.28). The age-specific trajectories in joy appeared to be largely stable, however, we still detected statistical evidence for a greater decline among the youngest compared with the older groups in 2017-2019 (e.g., age 14-25 vs >65: 0.08, 0.04 to 0.13). Moreover, while the frequency of joy was stable during the pandemic (2020-2021), it improved among the two oldest groups. Frequency of energy and optimism was the lowest among the youngest group and was steadily declining throughout the entire study period. However, we did not detect any statistical evidence for differences in change between the youngest and older age groups.

Among the indicators of negative affect, there was evidence for age differences in trends in depression and anxiety (p<0.0001), anger (p<0.0001), worry (p=0.004), and sadness (p=0.06). The youngest age group reported a greater increase in the feelings of depression and anxiety during pre-pandemic (2017-2019) (e.g., age 14-25 vs >65: 0.12, 0.05 to 0.19), and pandemic (2020-2021) (e.g., age 14-25 vs >65: 0.27, 0.11 to 0.43). Overall, they had the highest levels of feelings of depression and anxiety during most of the study period. The frequency of anger reduced among the youngest from 2017 to 2020, when it increased during pandemic and declined again out-of-pandemic. The key difference across age groups was that anger got worse to a greater extent during pandemic among the oldest group (i.e., age 14-25 vs >65: 0.18, 0.02 to 0.34), but then it also improved more (i.e., age 14-25 vs >65: 0.20, 0.03 to 0.37). The frequency of worry was the highest in the age 14-25 throughout the study period, and it increased to a greater extent in 2017-2019 than among the oldest individuals (e.g., age 14-25 vs >65: 0.08, 0.00 to 0.15). Finally, the trajectory of sadness was comparable across the age groups, with the oldest being sad at the highest frequency throughout the entire study period (2017-2023). The main difference between age group was that the youngest individuals reported a larger increase in anger between 2017 and 2019 than the oldest ones (i.e., age 14-25 vs >65: 0.08, 0.01 to 0.16).

Supplementary Figure 1. Age-specific average trajectories of individual indicators of wellbeing.

| Supplementary Table 1. P-values of Wald test of differential trajectories across population subgroups. | | | | | | |
| --- | --- | --- | --- | --- | --- | --- |
|  | PALS | NA | Stress | Sleeping problems | Headaches | Weakness |
| Period1719*gender | 0.48 | 0.29 | 0.44 | 0.96 | 0.70 | 0.86 |
| Period1920*gender | 0.67 | 0.04 | 0.73 | 0.94 | 0.69 | 0.55 |
| Period2021*gender | 0.36 | 0.04 | 0.65 | 0.50 | 0.46 | 0.90 |
| Period2122*gender | 0.20 | 0.53 | 0.82 | 0.54 | 0.09 | 0.44 |
| All periods*gender | 0.71 | 0.28 | 0.93 | 0.95 | 0.49 | 0.74 |
|  |  |  |  |  |  |  |
| Period1719*nationality | 0.10 | 0.77 | 0.67 | 0.55 | 0.21 | 0.50 |
| Period1920*nationality | 0.58 | 0.48 | 0.76 | 0.87 | 0.92 | 0.85 |
| Period2021*nationality | 0.56 | 0.93 | 0.71 | 0.61 | 0.37 | 0.69 |
| Period2122*nationality | 0.85 | 0.43 | 0.32 | 0.19 | 0.97 | 0.30 |
| All periods*nationality | 0.06 | 0.89 | 0.88 | 0.65 | 0.65 | 0.81 |
|  |  |  |  |  |  |  |
| Period1719*with parents | 0.13 | 0.97 | 0.31 | 0.30 | 0.52 | 0.51 |
| Period1920*with parents | 0.85 | 0.10 | 0.65 | 0.64 | 0.65 | 0.92 |
| Period2021*with parents | 0.57 | 0.10 | 0.76 | 0.83 | 0.69 | 0.69 |
| Period2122*with parents | 0.73 | 0.94 | 0.24 | 0.35 | 0.76 | 0.57 |
| All periods*with parents | 0.19 | 0.50 | 0.35 | 0.69 | 0.89 | 0.90 |
|  |  |  |  |  |  |  |
| Period1719*partner | 0.98 | 0.90 | 0.84 | 0.48 | 0.78 | 0.45 |
| Period1920*partner | 0.88 | 0.56 | 0.77 | 0.83 | 0.54 | 0.29 |
| Period2021*partner | 0.56 | 0.74 | 0.62 | 0.60 | 0.92 | 0.44 |
| Period2122*partner | 0.30 | 0.52 | 0.54 | 0.84 | 0.35 | 0.99 |
| All periods*partner | 0.39 | 0.91 | 0.98 | 0.92 | 0.85 | 0.85 |
|  |  |  |  |  |  |  |
| Period1719*education | 0.02 | 0.13 | 0.006 | 0.5146 | 0.17 | 0.96 |
| Period1920*education | 0.96 | 0.63 | 0.83 | 0.8288 | 0.33 | 0.77 |
| Period2021*education | 0.19 | 0.37 | 0.24 | 0.9640 | 0.64 | 0.97 |
| Period2122*education | 0.69 | 0.02 | 0.94 | 0.82 | 0.59 | 0.71 |
| All periods*education | 0.07 | 0.005 | 0.05 | 0.9878 | 0.63 | 0.98 |
|  |  |  |  |  |  |  |
| Period1719*income | 0.27 | 0.16 | 0.65 | 0.74 | 0.35 | 0.96 |
| Period1920*income | 0.49 | 0.31 | 0.69 | 0.79 | 0.44 | 0.84 |
| Period2021*income | 0.86 | 0.77 | 0.89 | 0.99 | 0.45 | 0.38 |
| Period2122*income | 0.70 | 0.22 | 0.96 | 0.65 | 0.99 | 0.87 |
| All periods*income | 0.91 | 0.35 | 0.99 | 0.94 | 0.80 | 0.95 |
| The table includes p values of Wald test that examines a null hypothesis of each interaction term equalling 0. | | | | | | |

**References**

1. Rothenbühler M, Voorpostel M. Attrition in the Swiss Household Panel: Are Vulnerable Groups more Affected than Others? In: Oris M, Roberts C, Joye D, Ernst Stähli M, editors. Surveying Human Vulnerabilities across the Life Course. Cham: Springer International Publishing; 2016. p. 223-44.

2. Collins LM, Schafer JL, Kam CM. A comparison of inclusive and restrictive strategies in modern missing data procedures. Psychological Methods. 2001;6(4):330-51.

3. Little R, Rubin DB. Statistical analysis with missing data. Hoboken, N.J: Wiley; 2002.

4. van Buuren S. Multiple imputation of discrete and continuous data by fully conditional specification. Stat Methods Med Res. 2007;16(3):219–42.

5. Sterne JA, White IR, Carlin JB, Spratt M, Royston P, Kenward MG, et al. Multiple imputation for missing data in epidemiological and clinical research: potential and pitfalls. BMJ. 2009;338:b2393.

6. Mostafa T, Narayanan M, Pongiglione B, Dodgeon B, Goodman A, Silverwood R, et al. Missing at random assumption made more plausible: evidence from the 1958 British birth cohort. Journal of Clinical Epidemiology. 2021.
